# Evaluation of newborn hearing screening program

**DOI:** 10.11606/s1518-8787.2020054001643

**Published:** 2020-04-23

**Authors:** Ana Carolina Alves Marinho, Edirlene Cordeiro de Souza Pereira, Kleyse Kerlyne Costa Torres, Andreza Monforte Miranda, Alleluia Lima Losno Ledesma

**Affiliations:** I Centro Universitário Planalto do Distrito FederalFaculdade de FonoaudiologiaBrasíliaDistrito Federal (DF)Brasil Centro Universitário Planalto do Distrito Federal (UNIPLAN). Faculdade de Fonoaudiologia . Brasília , Distrito Federal (DF) , Brasil; II Secretaria de Estado do Distrito FederalHospital Regional de SobradinhoBrasíliaDistrito Federal (DF)Brasil Secretaria de Estado do Distrito Federal (SES-DF). Hospital Regional de Sobradinho . Brasília , Distrito Federal (DF) . Brasil

**Keywords:** Newborn Screening, Hearing loss, congenital, Risk Factors, Evaluation of Health Programs and Projects

## Abstract

**OBJECTIVE:**

To evaluate Newborn Hearing Screening Program of Hospital Regional de Sobradinho, from January 2016 to December 2017, according to Multiprofessional Committee on Auditory Health parameters and Joint Committee on Infant Hearing (JCIH) recommendations, as well as to describe the prevalence of risk factors for hearing loss within the study population and their impact on the respective program.

**METHOD:**

This is a quantitative, cross-sectional and retrospective study that carefully analyzed registration books of screened newborns. It was established the prevalence of “pass” and “fail” in test and retest, retest percentage of attendance and referral for audiological diagnosis. Risk factors for hearing loss were described, as well as their influence on “pass” and “fail” rates. Inferential statistical analysis was performed using chi-square test and Anderson-Darling test, with 5% reliability index.

**RESULTS:**

A total of 3,981 newborns were screened; 2,963 (74.4%) presented no risk factors whereas 1,018 (25.6%) did, prematurity being the most frequent (51.6%). In the test, 166 (4.2%) failed and 118 (71.1%) attended the retest. The referral rate for diagnosis was 0.3%.

**CONCLUSION:**

Regarding the percentage of referral for diagnosis, the program reached indexes recommended by the Joint Committee on Infant Hearing and Multiprofessional Committee on Auditory Health. The most prevalent risk factor within the population was prematurity.

## INTRODUCTION

Universal Newborn Hearing Screening (UNHS) is part of a set of actions recommended by the Ministry of Health for integral care to hearing health in childhood., being responsible for the early detection of hearing loss in newborns through Otoacoustic emission (OAE) testing and automated brainstem auditory evoked potential (BAEP), also known as brainstem evoked response audiometry (BERA) ^1^ .

According to the Joint Committee on Infant Hearing ^2^ , OAE testing is indicated for the early identification of hearing disorders in newborns (NB). BAEP should be performed when, regardless of the OAE testing outcome, the newborn has any risk factor for hearing loss (RFHL): cytomegalovirus (CMV) infection, progressive hearing loss associate-syndromes, prematurity, neonatal intensive care unit (NICU) permanence, neurodegenerative disorders, trauma or postnatal infections with positive culture associated with sensorineural hearing loss, when small for gestational age (SGA), children who have received extracorporeal membrane oxygenation (ECMO) or chemotherapy, and whenever there is caregiver’s concern or hearing-loss family history ^2^ .

In 2010, the State sanctioned law No. 12,303 ^3^ , stating the compulsory free-execution of the examination called Evoked Otoacoustic Emissions (EOEA) within all hospitals and maternities in children born in their facilities. As the law neither set deadlines for compliance nor defined the funding sources, in 2012 the Ministry of Health established the UNHS Attention Guidelines in Brazil ^1^ . Literature agrees that UNHS execution rates should be higher than 95% of live births ^2 , 4^ . However, according to surveys, the reality of Brazil is far from this number, and, if hearing loss is not identified early, children may have great difficulties in speech and language development ^5^ .

Based on the above and on the reality of the Unified Health System, the need for discussions about the effectiveness of UNHS is reinforced. This study aims to evaluate the Newborn Hearing Screening Program of Hospital Regional de Sobradinho (HRS), from January 2016 to December 2017, according to Multiprofessional Committee on Auditory Health parameters and Joint Committee on Infant Hearing (JCIH) recommendations, as well as describe the prevalence of risk factors for hearing loss within the study population and their impact on the respective program. As research hypothesis, we assumed that the service could reach the values recommended by COMUSA and JCIH, although the population covered has a high prevalence of RFHL, and that the percentage of newborns with it affects the Program outcomes. Thus, this study is important as it contributes to other national newborn hearing screening programs on epidemiology, flow and main difficulties encountered in this population screening.

## METHODS

This is a quantitative, cross-sectional and retrospective study which carefully analyzed registration books of newborns screened between January 2016 and December 2017. It was performed in a public hospital, located in one of the four administrative regions that assemble the Northern Health Region of the Federal District. Hospital Regional de Sobradinho has an outpatient clinic with 31 specialties, emergency units and hospitalization clinics. Among them, units of maternal-child care: gynecology and obstetrics, obstetric center, maternity, neonatal intensive care unit, intermediate care unit and kangaroo care. In this area, it is a reference for high-risk pregnancies and deliveries. Access to inpatient units by regulation and emergency is open door ^6^ .

**Figure 1:**
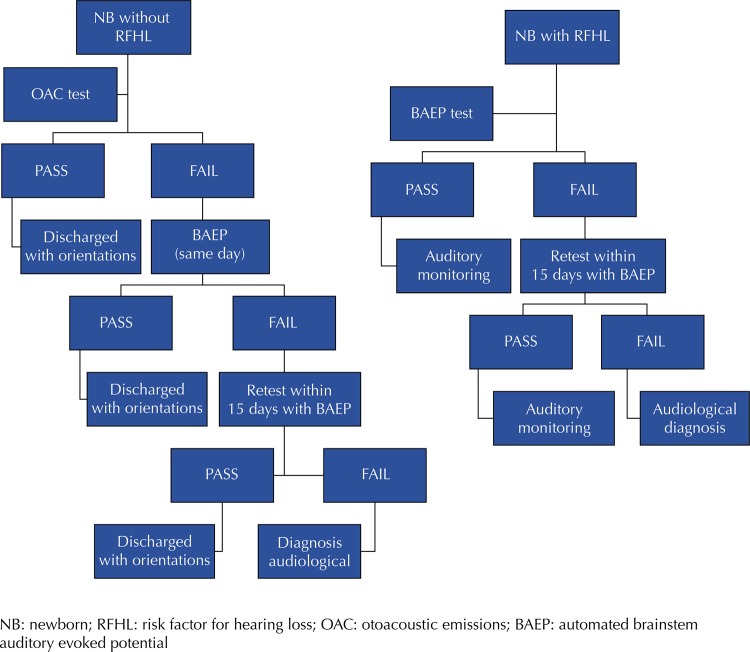
Newborn hearing screening adopted protocol.

The screening protocol of the program is ( Figure 1 ):

Transient Evoked Otoacoustic Emission (TEOAE) testings are performed by Otoread equipment. “Pass,” indicative of normal hearing, is established when minimum signal level response is greater than -12 dB, and minimum signal-to-noise ratio is 6 dB in at least 3 frequencies. BAEP exam is performed by Otometrics Accuscreen device at 35 dB, following “pass” and “fail” criteria established by the device.

As proposed by the Health Department of the Federal District, the following risk factors were evaluated: heredity, consanguinity, Intensive Care Unit (ICU) permanence, mechanical ventilation, ototoxic, hyperbilirubinemia to exchange transfusion (EXT) level, perinatal anoxia, Apgar 0 to 4 (in the first minute) and 0 to 6 (in the fifth minute), birth weight (BW) less than or equal to 1,500g, preterm newborn (PTNB), SGA, congenital infection, craniofacial anomalies, syndromes, postnatal infections and Down syndrome. The sample of live births was collected in the unit internal registry and compared with screened newborns number sample according to TEOAE and BAEP registration books.

The following data were collected from Newborn Hearing Screening program registration books: mother’s name, sex, date of examination, date of birth, RFHL and test result. Data were tabulated in Microsoft Excel: one spreadsheet for OAC testing, one for BAEP testing, and one for retesting (BAEP).

When aforementioned data were absent in the registration book, lacking information were searched in electronic medical records; records with non-existent information were excluded from the research. Data were collected using the physical book of the program because electronic medical-record research is more laborious, as it consists of a general record, with patient evolution in the service; this resource was reserved for cases in which there was incongruity in the physical record. All records were included in data analysis, except those in which information was still missing after electronic medical records analysis.

“Pass” and “fail” prevalence was established for each test based on collected data. Retesting attendance and diagnosis referral numbers were collected in BAEP registration books. From the raw data collected, percentages of each stage were established.

After data collection, chi-square test with 5% reliability index was used to perform an inferential statistical analysis, in order to calculate whether research results could be extrapolated to populations with the same parameters. For validation, Anderson-Darling test was performed to verify whether samples follow a normal distribution. Results were presented in tables and graphs.

The research followed recommendations on ethics in studies with human beings, approved by the Ethics Committee under CAEE 00620818.0.0000.5512.

## RESULTS

In 2016 and 2017, 3,981 newborns were screened at the Hospital Regional de Sobradinho; 1,992 in 2016 and 1,989 in 2017. Among these, 2,963 (74.4%) presented no RFHL, and 1,018 (25.6%) did. In the service records, there were 1,948 live births in 2016 and 1,932 in 2017, making up 102.3% of newborns screened in 2016 and 103.0% in 2017. Of the 3,981 screened newborns in 2016 and 2017, 166 (4.2%) failed the test, and 118 (71.1%) attended the retest. 12 (0.3%) failed the retest, being referred for audiological diagnosis.

In 2016, 1,519 NB without RFHL were screened, of which 1,454 (95.7%) passed and 65 (4.3%) failed. Among those who failed, 23 (35.4%) failed the right ear (RE), 25 (38.5%) left ear (LE) and 17 (26.1%) both ears. In 2017, 1,444 NB without RFHL were screened, of which 1,415 (98.0%) passed and 29 (2.0%) failed. Among those who failed, 9 (31.0%) failed RE, 15 (51.7%) LE and 5 (17.2%) both ears.

**Figure 2:**
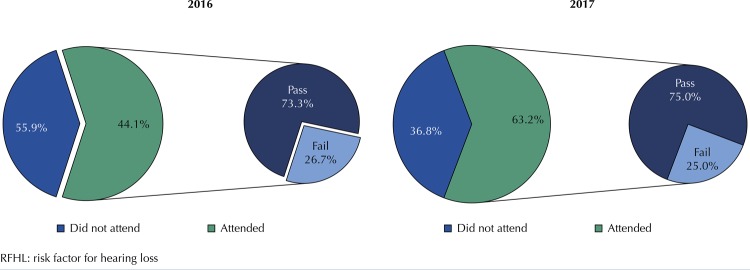
Prevalence of attendance, retest pass and fail of newborns without RFHL in the years 2016 and 2017.

In 2016, among newborns with no RFHL who failed, 56 (86%) attended retest, of which 55 (98.2%) passed and 1 (1.8%) failed. In 2017, 23 (79.3%) attended, of which 22 (95.7%) passed and 1 (4.3%) failed ( Figure 2 ).

In 2016, 473 NB without RFHL were screened, of which 439 (92.8%) passed and 34 (7.2%) failed. Among those who failed, 7 (20.6%) failed RE, 6 (17.6%) LE and 21 (61.8%) both ears. In 2017, 545 NB without RFHL were screened, of which 507 (93.0%) passed and 38 (7.0%) failed. Among those who failed, 4 (10.5%) failed RE, 20 (52.6%) LE and 14 (36.8%) both ears. There was no statistical significance between the failure percentage per ear within this population, with 0.8 p-value in 2016 and 0.1 in 2017.

**Figure 3:**
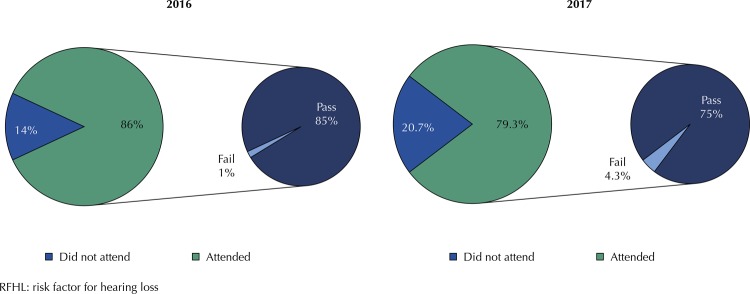
Prevalence of attendance, retest pass and fail of newborns with RFHL in the years 2016 and 2017.

In 2016, among newborns without RFHL who failed, 15 (44.1%) attended the retest, of which 11 (73.3%) passed and 4 (26.7%) failed. In 2017, 24 (63.2%) attended, of which 18 (75.0%) passed and 6 (25.0%) failed ( Figure 3 ).

A statistical relationship was observed between RFHL presence and the percentage of failure in newborn hearing screening, both in test and retest in 2016 and 2017 ( Table 1 ).

**Table 1:** Analysis of fail numbers and risk factors for hearing loss presence.

2016		Yes	No	Total		2017		Yes	No	Total
										
Test	Passed	439	1.454	1.893		Test	Passed	507	1.415	1.922
	Failed	34	65	99			Failed	38	29	67
	Total	473	1.519	1.992			Total	545	1.444	1.989
**Expected**						**Expected**				
**2016**		Yes	No	Total		**2017**		Yes	No	Total
**Test**	Passed	449.4925	1.443.508	1.893		**Test**	Passed	457.0669	1.467.832	1.893
	Failed	23.50753	75.49247	99			Failed	15.93313	51.16792	99
	Total	473	1.519	1.992			Total	473	1.519	1.992
**p = 0.0110**						**p** ≅ **0**				
										
**2016**		Yes	No	Total		**2017**		Yes	No	Total
**Retest 1**	Passed	11	55	66		**Retest 1**	Passed	11	55	66
	Failed	4	1	5			Failed	4	1	5
	Total	15	56	71			Total	15	56	71
**Expected**						**Expected**				
**2016**		Yes	No	Total		**2017**		Yes	No	Total
**Retest 1**	Passed	13.94366	52.05634	66		**Retest 1**	Passed	13.94366	52.05634	66
	Failed	1.056338	3.943662	5			Failed	1.056338	3.943662	5
	Total	15	56	71			Total	15	56	71
**p = 0.0008**						**p = 0.0008**	Chi-Square			

Table 2 shows RFHL prevalence within studied population: prematurity (PTNB) is the most prevalent, followed by ototoxic use and ICU permanence, both in 2016 and 2017.

**Table 2:** Prevalence of RFHL in screened live births at the Hospital Regional de Sobradinho in 2016 and 2017.

RFHL	Total	2016	2017
	n (%)	n (%)	n (%)
Heredity	123 (12.08%)	56 (11.8%)	67 (12.3%)
Consanguinity	44 (4.3%)	28 (5.9%)	16 (2.9%)
**ICU permanence**	**242 (23.8%)**	**110 (23.3%)**	**132 (24.2%)**
Mechanical Ventilation	119 (11.7%)	59 (12.5%)	60 (11.0%)
**Ototoxic**	**283 (27.8%)**	**136 (28.8%)**	**147 (27.0%)**
Hyperbilirubinemia	12 (1.2%)	7 (1.5%)	5 (0.9%)
Perinatal anoxia	8 (0.8%)	4 (0.8%)	4 (0.7%)
Apgar 0–4 / 0–6	112 (11.0%)	58 (12.3%)	54 (9.9%)
PN ≤ 1,500 g	93 (9.1%)	61 (12.9%)	32 (5.9%)
**PTNB**	**526 (51.6%)**	**256 (54.1%)**	**270 (49.5%)**
SGA	171 (16.7%)	78 (16.5%)	93 (17.1%)
Congenital infection	49 (4.8%)	19 (4.0%)	30 (5.5%)
Craniofacial anomalies	28 (2.7%)	7 (1.5%)	21 (3.9%)
Syndromes	5 (0.5%)	1 (0.2%)	4 (0.7%)
Postnatal infections	6 (0.6%)	3 (0.6%)	3 (0.6%)
Down syndrome	1 (0.1%)	1 (0.2%)	0 (0%)

RFHL: risk factor for hearing loss; ICU: intensive care unit; BW: birth weight; PTNB: preterm newborn; SGA: small for gestational age

Note: bold RFHL were the most prevalent

## DISCUSSION

JCIH recommends regular monitoring of Newborn Hearing Screening programs performance regarding coverage and “fail” percentages. As for program coverage, it can be inferred that it complies with COMUSA and JCHI recommendations of screening at least 95% of live births ^2 , 4^ . These results were also achieved by another newborn hearing screening program in Brazil ^7^ , while literature describes programs that did not achieve this rate ^8 , 9^ .

Screened newborns number exceeded that of live births. This can be justified because, according to Ordinance No. 1,459 of June 24, 2011, which establishes Stork Network, the program screens newborns from other hospitals who live in the regional, as well as newborns who, despite being born in other regions, were admitted and discharged from the hospital NICU ^6^ .

Test failure rate was comparable to that reported in another study, performed in four maternities in Paraná, that found 5%, 3% and 2% in three of the institutions (data were not presented in the last) ^10^ , but lower than presented in studies conducted in secondary-level maternities, which found 11.7% and 25.3% ^9 , 11^ .

Regarding failures laterality, higher prevalence was observed in the left ear; however, no statistical relevance was found. A previous study showed higher prevalence of right ear failure, with no statistical significance as well ^12^ . Another study showed a similar failure percentage in both ears ^13^ . As there is no literature consensus, it suggests no ear predominance in the percentage of hearing screening failures in newborns.

Retest attendance was 71.1%, similar to that obtained in another study, of approximately 75.7% ^14^ . Percentage of referrals for audiological diagnosis was 0.3%, compliant with JCHI and COMUSA resolution that this rate should not exceed 4% of screened newborns ^2 , 4^ . In a national study conducted in 2017, 6.02% of screened newborns were referred for audiological diagnosis ^15^ , whereas this rate was 1.7% in another study ^9^ .

As 2016 retest non-attendance rate among NB with RFHL (55.9%) exceeded that of 2017 (20.7%), it can be suggested that the UNHS program of the studied hospital reinforced guidelines on the importance of retest attendance. This is an orientation of paramount importance, as retest non-attendance delays diagnosis on probable hearing loss, making it impossible to minimize damage to language development. Lack of knowledge and understanding on the importance of auditory examination may interfere directly in early deafness identification ^16^ .

As for hearing loss risks, prematurity was observed to be the most prevalent, corroborating a study conducted at Hospital Universitario de Santa Maria ^17^ , and differing from another, in which ICU permanence over five days was the most observed RFHL, and prematurity was the second one ^18^ . Data differed from a study conducted in Maceió, in which hyperbilirubinemia was the most frequent risk factor; however, prematurity was not included among RFHL ^19^ . It is worth noting that JHIC does not refer to prematurity as a risk factor for hearing loss when compared in isolation ^2^ . This index inclusion may be justified by the fact that PTNB have a higher risk of biological alteration in global development, what may interfere in the auditory pathway maturation in a harmful way ^20^ .

In the study population, of 3,981 screened neonates, 1,018 (25.6%) presented one or more risk factors for hearing loss. In a survey conducted with 1,570 NB, 221 (14.1%) presented one or more RFHL ^9^ . In another study, the sample was made up of 1,626 newborns, of which 163 (10.0%) presented one or more RFHL ^19^ . This study reported a higher prevalence of newborns with RFHL when compared to studies aforementioned. According to Ordinance No. 47 of March 13, 2014, HRS is considered a reference for high-risk deliveries to a few neighboring municipalities, justifying the high rate of newborns with RFHL within this population, thus increasing the prevalence of RFHL in newborns ^21^ .

As limitations, this study did not investigate demographic factors influence and type of delivery on hearing screening results; moreover, it was impossible to define the percentage of coverage of screened newborns, due to the lack of information on their birthplace.

The program reached JCIH and COMUSA recommended rates on diagnosis referrals, despite RFHL high rate within the study population. As for coverage, it was not possible to affirm that the program complies with these committees’ recommendations. Most prevalent RFHL within the population was prematurity, followed by ototoxic use and ICU permanence.
