# Supplementary material for: Evaluation of newborn hearing screening program
Source: Rev Saude Publica. 2020 Apr 23;54:44. doi: 10.11606/s1518-8787.2020054001643 (PMC7185987; doi:10.11606/s1518-8787.2020054001643)
Supplement: Translation

# Translation: Avaliação de um programa de triagem auditiva neonatal

**Authors:** Ana Carolina Alves Marinho, Edirlene Cordeiro de Souza Pereira, Kleyse Kerlyne Costa Torres, Andreza Monforte Miranda, Alleluia Lima Losno Ledesma

**Affiliations:** I BrasíliaDistrito Federal (DF)Brasil Centro Universitário Planalto do Distrito Federal (UNIPLAN). Faculdade de Fonoaudiologia. Brasília , Distrito Federal (DF) , Brasil; II BrasíliaDistrito Federal (DF)Brasil Secretaria de Estado do Distrito Federal (SES-DF). Hospital Regional de Sobradinho. Brasília , Distrito Federal (DF) . Brasil

**Keywords:** Triagem Neonatal, Perda Auditiva, congênito, Fatores de Risco, Avaliação de Programas e Projetos de Saúde

## Abstract

**OBJETIVO:**

Avaliar o Programa de Triagem Auditiva Neonatal do Hospital Regional de Sobradinho, no período de janeiro de 2016 a dezembro de 2017, segundo os parâmetros do Comitê Multiprofissional em Saúde Auditiva e as recomendações do *Joint Committee on Infant Hearing* (JCIH), bem como descrever a prevalência dos indicadores de risco para deficiência auditiva na população estudada e seu impacto no respectivo programa.

**MÉTODO:**

Trata-se de um estudo quantitativo, transversal e retrospectivo no qual foram analisados criteriosamente livros de registros dos neonatos triados. Foi estabelecida a prevalência de “passa” e “falha” no teste e reteste, o percentual de comparecimento para reteste e de encaminhamento para diagnóstico audiológico. Foram descritos os indicadores de risco para deficiência auditiva, bem como sua influência nos índices de “passa” e “falha”. Foi realizada análise estatística inferencial utilizando o teste do qui-quadrado e o teste de Anderson-Darling, com índice de confiabilidade de 5%.

**RESULTADOS:**

Foram triados 3.981 neonatos, 2.963 (74,4%) dos quais sem indicadores de risco e 1.018 (25,6%) com, sendo a prematuridade o mais frequente (51,6%). No teste, 166 (4,2%) falharam e 118 (71,1%) compareceram para o reteste. O índice de encaminhamento para diagnóstico foi de 0,3%.

**CONCLUSÃO:**

O programa atingiu os índices recomendados pelo *Joint Committee on Infant Hearing* e pelo Comitê Multiprofissional em Saúde Auditiva quanto à porcentagem de encaminhamento para diagnóstico. O indicador de risco mais prevalente na população foi a prematuridade.

## INTRODUÇÃO

A triagem auditiva neonatal universal (TANU) faz parte de um conjunto de ações preconizadas pelo Ministério da Saúde para a atenção integral à saúde auditiva na infância, sendo responsável pela detecção precoce de perda auditiva em neonatos por meio dos exames de emissões otoacústicas (EOA) e potencial evocado auditivo de tronco encefálico automático (PEATE-A), conhecido também como brainstem evoked response audiometry (BERA) ^1^ .

De acordo com o *Joint Committee on Infant Hearing* ^2^ , é indicada a realização do exame de EOA para identificação precoce de alterações auditivas em recém-nascidos (RN). O PEATE-A deve ser realizado quando, independentemente do resultado do exame de EOA, o recém-nascido possuir algum indicador de risco para deficiência auditiva (IRDA): infecção por citomegalovírus (CMV), síndromes associadas à perda auditiva progressiva, prematuridade, permanência na unidade de terapia intensiva neonatal (UTIN), distúrbios neurodegenerativos, trauma ou infecções pós-natais com cultura positiva associadas à perda auditiva sensorioneural, quando pequeno para a idade gestacional (PIG), para crianças que receberam oxigenação por membrana extracorpórea (ECMO) ou quimioterapia e quando há preocupação do cuidador ou história familiar de perda auditiva ^2^ .

Em 2010, foi sancionada a lei n° 12.303 ^3^ , que dispõe sobre a obrigatoriedade de realização gratuita do exame denominado emissões otoacústicas evocadas – conhecido também como “teste da orelhinha” – em todos os hospitais e maternidades, nas crianças nascidas em suas dependências. Visto que a lei não estabeleceu prazos para cumprimento nem definiu as fontes de financiamento, em 2012 o Ministério da Saúde instaurou as Diretrizes de Atenção da TANU no Brasil ^1^ . A literatura entra em consenso de que os índices de realização da TANU devem ser superiores a 95% dos nascidos vivos ^2 , 4^ . Entretanto, de acordo com levantamentos realizados, a realidade do Brasil está distante desse número, fato preocupante, uma vez que, caso a perda auditiva não seja identificada precocemente, essa criança poderá ter grandes dificuldades no desenvolvimento da fala e da linguagem ^5^ .

A partir da afirmativa acima e da realidade do Sistema Único de Saúde, reforça-se a necessidade de discussões acerca da efetividade da TANU. Este estudo tem como objetivo avaliar o Programa de Triagem Auditiva Neonatal do Hospital Regional de Sobradinho (HRS), no período de janeiro de 2016 a dezembro de 2017, segundo os parâmetros do Comitê Multiprofissional em Saúde Auditiva (Comusa) e as recomendações do *Joint Committee on Infant Hearing* (JCIH), bem como descrever a prevalência dos indicadores de risco para deficiência auditiva na população investigada e seu impacto no respectivo programa. Como hipótese de pesquisa, supõe-se que o serviço consegue alcançar os valores preconizados pelo Comusa e JCIH, apesar de a população atendida possuir elevada prevalência de IRDA, e que o percentual de recém-nascidos com IRDA impacta os resultados alcançados pelo programa. Assim, este estudo adquire importância por fornecer contribuição para os demais programas de triagem auditiva neonatais nacionais sobre a epidemiologia, o fluxo e principais dificuldades encontradas na triagem desta população.

## MÉTODOS

Trata-se de um estudo quantitativo, transversal e retrospectivo, no qual foram analisados criteriosamente livros de registros dos neonatos que foram triados entre janeiro de 2016 e dezembro de 2017. Foi realizado em um hospital público situado em uma das quatro regiões administrativas que compõem a Região de Saúde Norte do Distrito Federal. O Hospital Regional de Sobradinho conta com ambulatório com 31 especialidades, unidades de emergência e clínicas de internação, dentre elas as seguintes unidades de atenção materno-infantil: ginecologia e obstetrícia, centro obstétrico, maternidade, unidade de terapia intensiva neonatal, unidade de cuidados intermediários convencional e canguru. Nessa área, ele é referência para as gestações e partos de alto risco, sendo o acesso para as unidades de internação via regulação e para a emergência, porta aberta ^6^ .

O programa tem como protocolo de triagem ( Figura 1 ):

**Figura 1:** Protocolo de triagem auditiva neonatal adotado.

Os exames de emissões otoacústicas transientes (EOAT) são realizados com o equipamento Otoread. É considerado “passa”, ou seja, indicativo de audição com normalidade, quando o nível mínimo de sinal apresentar resposta superior a -12 dB e a relação sinal-ruído for de no mínimo 6 dB em pelo menos 3 frequências. O exame de PEATE-A é realizado com o equipamento Acuscreen da Otometrics a 35 dB, seguindo os critérios de “passa” e “falha” estabelecidos pelo equipamento.

Foram avaliados os seguintes indicadores de risco, conforme proposto pela Secretaria de Saúde do Distrito Federal: hereditariedade, consanguinidade, permanência na unidade de terapia intensiva (UTI), ventilação mecânica, ototóxico, hiperbilirrubinemia a nível de exsanguineotransfusão, anóxia perinatal, Apgar 0 a 4 (no primeiro minuto) e 0 a 6 (no quinto minuto), peso ao nascer (PN) menor ou igual a 1.500g, recém-nascido pré-termo (RNPT), PIG, infecção congênita, anomalias craniofaciais, síndromes, infecções pós-natais e síndrome de Down. A amostra da quantidade de nascidos vivos foi coletada em registro interno da unidade e comparada com a amostra da quantidade de neonatos triados segundo os livros de registro de EOAT e PEATE-A.

Foram coletados nos livros de registro do Programa de Triagem Auditiva Neonatal os seguintes dados: nome da mãe, sexo, data do exame, data de nascimento, IRDA e resultado do exame realizado. Os dados foram organizados em planilhas de Microsoft Excel, sendo uma planilha para teste com EOA, uma para teste com PEATE-A, e uma para reteste (PEATE-A).

Na ausência dos dados supracitados, foi realizada busca no prontuário eletrônico pelas informações inexistentes no livro de registro, sendo excluídos da pesquisa os registros com qualquer uma das informações inexistentes. A coleta dos dados deu-se no livro físico do programa pois a pesquisa no prontuário eletrônico é mais laboriosa, por ser um registro geral com toda a evolução do paciente no serviço, sendo reservado esse recurso apenas para os casos onde houvesse alguma incongruência no registro físico. Todos os registros entraram na análise dos dados, sendo excluídos aqueles nos quais faltasse alguma das informações após a análise do prontuário eletrônico.

Foi estabelecida a prevalência de “passa” e “falha” em cada um dos testes com base nesses dados coletados. A quantidade de comparecimentos para reteste bem como de encaminhamentos para diagnóstico foi coletada nos livros de registro do PEATE-A. A partir dos dados brutos coletados, foram estabelecidos os percentuais de cada etapa.

Após o levantamento de dados, foi realizada análise estatística inferencial para calcular se os resultados da pesquisa podem ser extrapolados para populações com os mesmos parâmetros desta, utilizando o teste do qui-quadrado com índice de confiabilidade de 5%. Para a validação desse teste, foi feito o teste de Anderson-Darling para verificar se as amostras seguem uma distribuição normal. Os resultados foram apresentados em tabelas e gráficos.

A pesquisa seguiu as recomendações sobre ética em estudos com seres humanos, sendo aprovado pelo Comitê de Ética sob o CAEE 00620818.0.0000.5512.

## RESULTADOS

Foram triados no Hospital Regional de Sobradinho, nos anos de 2016 e 2017, 3.981 recém-nascidos, sendo 1.992 em 2016 e 1.989 em 2017. Desses, 2.963 (74,4%) não tinham IRDA e 1.018 (25,6%) tinham. Nos registros do referido serviço, observaram-se 1.948 nascidos vivos em 2016 e 1.932 nascidos vivos em 2017, perfazendo 102,3% de recém-nascidos triados em 2016 e 103,0% em 2017. Dos 3.981 neonatos triados nos anos de 2016 e 2017, 166 (4,2%) falharam no teste, sendo que 118 (71,1%) compareceram para o reteste. Desses, 12 (0,3%) falharam no reteste e foram encaminhados para diagnóstico audiológico.

No ano de 2016, foram triados 1.519 RN sem IRDA, dos quais 1.454 (95,7%) passaram e 65 (4,3%) falharam. Dentre os que falharam, 23 (35,4%) falharam na orelha direita (OD), 25 (38,5%) na orelha esquerda (OE) e 17 (26,1%) em ambas as orelhas. No ano de 2017, foram triados 1.444 RN sem IRDA, dos quais 1.415 (98,0%) passaram e 29 (2,0%) falharam. Dentre os que falharam, 9 (31,0%) falharam na OD, 15 (51,7%) na OE e 5 (17,2%) em ambas as orelhas.

No ano de 2016, dos neonatos sem IRDA que falharam, 56 (86%) compareceram para reteste, dos quais 55 (98,2%) passaram e 1 (1,8%) falhou. No ano de 2017, 23 (79,3%) compareceram, dos quais 22 (95,7%) passaram e 1 (4,3%) falhou ( Figura 2 ).

**Figura 2:** Prevalência de comparecimento, passa e falha no reteste de recém-nascidos sem IRDA nos anos de 2016 e 2017.

No ano de 2016 foram triados 473 RN com IRDA, dos quais 439 (92,8%) passaram e 34 (7,2%) falharam. Dentre os que falharam, 7 (20,6%) falharam na OD, 6 (17,6%) na OE e 21 (61,8%) em ambas as orelhas. No ano de 2017 foram triados 545 RN com IRDA, dos quais 507 (93,0%) passaram e 38 (7,0%) falharam. Dentre os que falharam, 4 (10,5%) falharam na OD, 20 (52,6%) na OE e 14 (36,8%) em ambas as orelhas. Não houve significância estatística entre o percentual de falhas por orelha nessa população, com p-valor de 0,8 e 0,1, respectivamente em 2016 e 2017.

No ano de 2016, dos neonatos com IRDA que falharam, 15 (44,1%) compareceram para reteste, dos quais 11 (73,3%) passaram e 4 (26,7%) falharam. No ano de 2017, 24 (63,2%) compareceram, dos quais 18 (75,0%) passaram e 6 (25,0%) falharam ( Figura 3 ).

**Figura 3:** Prevalência de comparecimento, passa e falha no reteste de recém-nascidos com IRDA nos anos de 2016 e 2017.

Foi observada relação estatística entre a presença de IRDA e o percentual de falha na triagem auditiva neonatal tanto no teste quanto no reteste nos anos de 2016 e de 2017 ( Tabela 1 ).

**Tabela 1:** Análise do número de falhas e da presença de indicadores de risco para deficiência auditiva.

2016		Sim	Não	Total		2017		Sim	Não	Total
Teste	Passaram	439	1.454	1.893		Teste	Passaram	507	1.415	1.922
	Falharam	34	65	99			Falharam	38	29	67
	Total	473	1.519	1.992			Total	545	1.444	1.989
**Esperada**						**Esperada**				
**2016**		Sim	Não	Total		**2017**		Sim	Não	Total
**Teste**	Passaram	449,4925	1.443,508	1.893		**Teste**	Passaram	457,0669	1.467,832	1.893
	Falharam	23,50753	75,49247	99			Falharam	15,93313	51,16792	99
	Total	473	1.519	1.992			Total	473	1.519	1.992
**p = 0,0110**						**p ≅ 0**				
										
**2016**		Sim	Não	Total		**2017**		Sim	Não	Total
**Reteste**	Passaram	11	55	66		**Reteste**	Passaram	11	55	66
	Falharam	4	1	5			Falharam	4	1	5
	Total	15	56	71			Total	15	56	71
**Esperada**						**Esperada**				
**2016**		Sim	Não	Total		**2017**		Sim	Não	Total
**Reteste**	Passaram	13,94366	52,05634	66		**Reteste**	Passaram	13,94366	52,05634	66
	Falharam	1,056338	3,943662	5			Falharam	1,056338	3,943662	5
	Total	15	56	71			Total	15	56	71
**p = 0,0008**						**p = 0,0008**	Qui-quadrado			

A Tabela 2 apresenta a prevalência dos IRDA na população estudada, sendo a prematuridade (RNPT) o mais prevalente, seguido do uso de ototóxico e da permanência na UTI, tanto no ano de 2016 quanto em 2017.

**Tabela 2:** Prevalência de IRDA em neonatos vivos triados no Hospital Regional de Sobradinho em 2016 e 2017.

IRDA	Total	2016	2017
	n (%)	n (%)	n (%)
Hereditariedade	123 (12,08%)	56 (11,8%)	67 (12,3%)
Consanguinidade	44 (4,3%)	28 (5,9%)	16 (2,9%)
**Permanência na UTI**	**242 (23,8%)**	**110 (23,3%)**	**132 (24,2%)**
Ventilação mecânica	119 (11,7%)	59 (12,5%)	60 (11,0%)
**Ototóxico**	**283 (27,8%)**	**136 (28,8%)**	**147 (27,0%)**
Hiperbilirrubinemia	12 (1,2%)	7 (1,5%)	5 (0,9%)
Anóxia perinatal	8 (0,8%)	4 (0,8%)	4 (0,7%)
Apgar 0–4 / 0–6	112 (11,0%)	58 (12,3%)	54 (9,9%)
PN ≤ 1.500 g	93 (9,1%)	61 (12,9%)	32 (5,9%)
**RNPT**	**526 (51,6%)**	**256 (54,1%)**	**270 (49,5%)**
PIG	171 (16,7%)	78 (16,5%)	93 (17,1%)
Infecção congênita	49 (4,8%)	19 (4,0%)	30 (5,5%)
Anomalias craniofaciais	28 (2,7%)	7 (1,5%)	21 (3,9%)
Síndromes	5 (0,5%)	1 (0,2%)	4 (0,7%)
Infecções pós-natais	6 (0,6%)	3 (0,6%)	3 (0,6%)
Síndrome de Down	1 (0,1%)	1 (0,2%)	0 (0%)

IRDA: indicador de risco para deficiência auditiva; UTI: unidade de terapia intensiva; PN: peso ao nascer; RNPT: recém-nascido pré-termo; PIG: pequeno para a idade gestacional

Nota: os IRDA em negrito foram os mais prevalentes

## DISCUSSÃO

O JCIH recomenda o monitoramento regular do desempenho dos programas de triagem auditiva neonatal em relação à cobertura e percentuais de “falha”. Tomando como base a cobertura do referido programa, pode-se inferir que ele obedece às recomendações do Comusa e do JCHI de que pelo menos 95% dos neonatos vivos sejam triados ^2 , 4^ . Esses resultados também foram alcançados por outro programa de triagem auditiva neonatal no Brasil ^7^ , enquanto a literatura descreve programas que não alcançaram esse índice ^8 , 9^ .

O número de neonatos triados superou o número de nascidos vivos. Isso pode ser justificado pois, conforme a portaria n°1.459 de 24 de junho de 2011, que institui a Rede Cegonha, o programa realiza a triagem de recém-nascidos provenientes de outros hospitais e que residam na mesma regional, bem como dos recém-nascidos que, apesar de nascidos em outras regionais, foram internados na UTIN desse hospital, recebendo alta na instituição ^6^ *.*

O índice de falha no teste foi comparável ao relatado em outro estudo que o pesquisou em quatro maternidades no Paraná, encontrando 5%, 3% e 2% em três das instituições (não foram apresentados esses dados na última) ^10^ . Foi inferior ao de estudos realizados em maternidades de nível secundário, que encontraram 11,7% e 25,3% ^9 , 11^ .

Em relação à lateralidade das falhas, foi observada uma maior prevalência na orelha esquerda; no entanto, não houve relevância estatística para esse achado. Estudo anterior demonstrou maior prevalência de falha na orelha direita também com ausência de significância estatística ^12^ , outro ainda demonstrou percentual de falha semelhante em ambas as orelhas ^13^ . Como não há consenso na literatura, sugere-se não haver predominância de orelhas no percentual de falhas na triagem auditiva em neonatos.

O comparecimento para reteste foi de 71,1%, índice semelhante ao obtido em outro estudo, de aproximadamente 75,7% ^14^ . A porcentagem de encaminhados para diagnóstico audiológico foi de 0,3%, o que está de acordo com a porcentagem indicada pelo JCHI e pelo Comusa, que determinam que esse índice não deve ultrapassar 4% dos neonatos triados ^2 , 4^ . Em um estudo nacional realizado em 2017, 6,02% dos neonatos triados foram encaminhados para diagnóstico audiológico ^15^ , enquanto em outro estudo essa taxa foi de 1,7% ^9^ .

Visto que no ano de 2016 o índice de não comparecimento para o reteste entre os RN com IRDA (55,9%) superou o do ano de 2017 (20,7%), pode-se sugerir que o programa da TANU do hospital estudado reforçou as orientações sobre a importância do comparecimento para o reteste. Essa orientação é de suma importância, considerando que o não comparecimento para o reteste atrasa o diagnóstico de prováveis perdas auditivas, não sendo possível minimizar prejuízos ao desenvolvimento da linguagem. Pode-se destacar que a falta de conhecimento ou até mesmo de compreensão em relação à importância do exame auditivo pode interferir diretamente na identificação precoce da surdez ^16^ .

Em relação aos riscos para deficiência auditiva, foi observado que a prematuridade foi o de maior prevalência, corroborando um estudo realizado no Hospital Universitário de Santa Maria ^17^ e diferindo de um estudo em que a permanência na UTI por mais de cinco dias foi o IRDA mais observado, porém no qual a prematuridade foi o segundo indicador mais prevalente ^18^ . Os dados divergiram também de um estudo realizado em Maceió, o qual obteve a hiperbilirrubinemia como indicador de risco mais frequente; no entanto, a prematuridade não foi incluída entre os IRDA ^19^ . Cabe destacar que o JHIC não faz referência à prematuridade como um fator de risco para deficiência auditiva se comparado isoladamente ^2^ . A inclusão desse indicador pode ser justificada pelo fato de os RNPT possuírem um maior risco de alteração biológica no desenvolvimento global, podendo interferir na maturação da via auditiva de maneira nociva ^20^ .

Na população estudada, dos 3.981 neonatos triados, 1.018 (25,6%) apresentaram um ou mais indicadores de risco para deficiência auditiva. Em uma pesquisa realizada com 1.570 RN, 221 (14,1%) apresentaram um ou mais IRDA ^9^ . Em outro estudo, a população estudada foi de 1.626 neonatos, dos quais 163 (10,0%) apresentaram um ou mais IRDA ^19^ . O presente estudo apresenta prevalência superior de RN com IRDA quando comparado aos estudos referidos. De acordo com a portaria n° 47 de 13 de março de 2014, o HRS é considerado referência para partos de alto risco em um grande número de municípios vizinhos, justificando o elevado índice de neonatos com IRDA nesta população, elevando assim a prevalência de IRDA nos neonatos ^21^ .

Como limitações, este estudo não investigou a influência de fatores demográficos e do tipo de parto nos resultados da triagem auditiva; além disso, foi impossível definir a porcentagem da cobertura de recém-nascidos triados, devido à inexistência da informação do local de nascimento do neonato.

O programa atingiu os índices recomendados pelo JCIH e pelo Comusa quanto à porcentagem de encaminhamento para diagnóstico, apesar do elevado índice de IRDA na população estudada. Não foi possível afirmar que o programa obedece às recomendações desses comitês quanto à cobertura. O IRDA mais prevalente na população foi a prematuridade, seguido de uso de ototóxico e permanência na UTIN.
